# Prevalence and familial patterns of gastrointestinal symptoms, joint hypermobility and diurnal blood pressure variations in patients with anorexia nervosa

**DOI:** 10.1186/2050-2974-1-S1-O45

**Published:** 2013-11-14

**Authors:** Min Goh, James Olver, Chia Huang, Melinda Millard, Chris O'Callaghan

**Affiliations:** 1 Department of Clinical Pharmacology, Austin Health, Australia; 2 University of Melbourne, Australia; 3 The Melbourne Clinic, Australia; 4 Victorian Spinal Cord Service, Austin Health, Australia

## 

Anorexia nervosa (AN) is associated with gut and non-gut symptoms e.g. orthostatic intolerance and fatigue syndromes. Elsewhere, joint hypermobility has been found in non-anorexic populations who have gut symptoms, orthostatic intolerance or fatigue syndromes. We therefore postulated that joint hypermobility may be a feature of AN.Patients from an inpatient Eating Disorders Unit and their first degree relatives underwent medical interview, joint mobility assessment, postural and ambulatory blood pressure (BP) measurements and completed a number of questionnaires. Comparisons were made with healthy sex matched controls.

Patients (n=30), relatives (n=29) and controls (n=16) were aged 24.4±1.4 years (mean ± SEM), 44.7±2.9 years and 38.6±3.7 years respectively. Joint hypermobility was more common in patients (63%) than relatives (34%) and controls (13%). Gastrointestinal or orthostatic intolerance symptoms prior to onset of AN were reported by 8 and 14 patients respectively. Orthostatic tachycardia, orthostatic hypotension and blunted diurnal BP pattern were found in 58%, 21% and 50% of patients respectively.

Patients with AN are characterised by a high prevalence of gut symptoms, orthostatic intolerance, and joint hypermobility. First degree relatives also have these complaints. No causal mechanism has been identified, but we hypothesise that the joint hypermobility is a possible indicator of a familial disorder of connective tissue elasticity which potentially plays a causal role in the development of the eating disorder.

This abstract was presented in the **Anorexia Nervosa – Characteristics and Treatment** stream of the 2013 ANZAED Conference.

